# Distribution and Prevalence of the Australian Non-Pathogenic Rabbit Calicivirus Is Correlated with Rainfall and Temperature

**DOI:** 10.1371/journal.pone.0113976

**Published:** 2014-12-08

**Authors:** June Liu, Damien A. Fordham, Brian D. Cooke, Tarnya Cox, Greg Mutze, Tanja Strive

**Affiliations:** 1 Commonwealth Scientific and Industrial Research Organisation, Ecosystem Sciences Division, Canberra, Australian Capital Territory 2601, Australia; 2 The Environment Institute and School of Earth and Environmental Sciences, University of Adelaide, Adelaide, South Australia 5005, Australia; 3 Institute of Applied Ecology, University of Canberra, Canberra, Australian Capital Territory 2601, Australia; 4 Invasive Animals Cooperative Research Centre, University of Canberra, Canberra, Australian Capital Territory 2601, Australia; 5 Vertebrate Pest Research Unit, NSW Department Primary Industries, Orange, New South Wales 2800, Australia; 6 Natural Resources Management Biosecurity Unit, Department of Water, Land and Biodiversity Conservation, Adelaide, South Australia 5001, Australia; University of New South Wales, Australia

## Abstract

**Background:**

Australia relies heavily on rabbit haemorrhagic disease virus (RHDV) for the biological control of introduced European wild rabbits *Oryctolagus cuniculus*, which are significant economic and environmental pests. An endemic non-pathogenic rabbit calicivirus termed RCV–A1 also occurs in wild rabbits in Australian and provides partial protection against lethal RHDV infection, thus interfering with effective rabbit control. Despite its obvious importance for rabbit population management, little is known about the epidemiology of this benign rabbit calicivirus.

**Methods:**

We determined the continent-wide distribution and prevalence of RCV-A1 by analysing 1,805 serum samples from wild rabbit populations at 78 sites across Australia for the presence of antibodies to RCV-A1 using a serological test that specifically detects RCV-A1 antibodies and does not cross-react with co-occurring RHDV antibodies. We also investigated possible correlation between climate variables and prevalence of RCV-A1 by using generalised linear mixed effect models.

**Results:**

Antibodies to RCV-A1 were predominantly detected in rabbit populations in cool, high rainfall areas of the south-east and south-west of the continent. There was strong support for modelling RCV-A1 prevalence as a function of average annual rainfall and minimum temperature. The best ranked model explained 26% of the model structural deviance. According to this model, distribution and prevalence of RCV-A1 is positively correlated with periods of above average rainfall and negatively correlated with periods of drought.

**Implications:**

Our statistical model of RCV-A1 prevalence will greatly increase our understanding of RCV-A1 epidemiology and its interaction with RHDV in Australia. By defining the environmental conditions associated with the prevalence of RCV-A1, it also contributes towards understanding the distribution of similar viruses in New Zealand and Europe.

## Introduction

As one of the most successful invasive animals world-wide, European wild rabbits (*Oryctolagus cuniculus*) have long impaired the economy and environment in Australia and some areas of New Zealand [1]. They reduce native pasture biodiversity, promote invasive weed species, restrict the regeneration of trees and shrubs, and have severe impacts on native herbivores and omnivores through food competition and habitat degradation, costing over AU$200 million in lost agricultural production each year in Australia [2].

Effective biological control of rabbits in Australia was initiated with the introduction of myxoma virus in the early 1950s and was followed with the release of rabbit haemorrhagic disease virus (RHDV) in 1995 [3]–[5]. Both viruses showed very different characteristics. Transmission of myxoma virus relies on biting insects such as fleas and mosquitoes, and therefore the virus spreads more effectively along waterways and in more humid climates that support mosquito abundance [6]. In contrast, the effect of RHDV on reducing rabbit abundance was found to be more severe in hot, dry areas compared to cool, wet areas [7], [8].

A large proportion of rabbits sampled in cool and wet areas in Australia had antibodies which cross reacted with RHDV in serological tests even though they were not exposed to RHDV previously. When rabbits from these areas were experimentally infected with RHDV, the case fatality rate was reduced by up to 52%. Thus it was hypothesised that the reduced impact of RHDV in cool and wet areas of Australia was due to a related, non-pathogenic rabbit calicivirus already present in wild rabbit populations that was providing some degree of cross protective immunity to lethal RHDV infection [7], [9]–[12]. Cross reacting antibodies in rabbits before the arrival of RHDV were also reported in New Zealand [13].

The benign rabbit calicivirus RCV-A1 was isolated and formally described in 2009 [14]. Subsequent studies showed that pre-infection with RCV-A1 can provide rabbits with up to 50% protection against lethal RHDV infection [15]. Notably, the cross protection is transient and wanes approximately 8 weeks following RCV-A1 infection [16]. Phylogenetic studies of different strains of RCV-A1 revealed that RCV-A1 had probably arrived in Australia together with the first wild rabbits approximately 150 years ago [17], and had probably reached the natural limits of its distribution in Australia well before RHDV was introduced. Following its initial release, RHDV spread through almost the entire rabbit population across Australia, yet a reduction of its effectiveness was only observed in the cooler, wetter areas. This indicated that if RCV-A1 was the underlying cause of the reduced impact of RHDV, its distribution may be regionally limited [7], [18].

As RCV-A1 and RHDV are closely related and antibodies raised against each virus are cross-reactive, the omnipresence of RHDV made it very difficult to determine the actual distribution of RCV-A1. However, following the discovery and genetic characterisation of RCV-A1 highly specific and sensitive enzyme-linked immunosorbent assays (ELISAs) could be developed [19], [20]. These assays specifically detect RCV-A1 antibodies at an individual level and, for the first time, allow the estimation of RCV-A1 prevalence in rabbit populations that also carry RHDV antibodies.

We applied these specific assays to 1805 recent and archival rabbit serum samples from 78 sites across Australia to determine presence and prevalence of RCV-A1, and subsequently used generalised linear mixed-effect models to test whether RCV-A1 distribution and prevalence can be explained by climatic conditions. We mapped the spatial and temporal variations of RCV-A1 prevalence in Australia, and discuss the implications for rabbit management.

## Materials and Methods

### Serum samples and serological assay

Rabbit serum samples collected from 78 sites across Australia between 1972 and 2012 for various rabbit research projects (e.g. to monitor the initial spread of RHDV) were used for detection of RCV-A1 antibodies (S1 Table). The sample size varied from 6 to 66 across sites.

Rabbit sera were tested by using a previously described competition ELISA that specifically detects RCV-A1 antibodies and does not cross react with RHDV antibodies (RCV-A1 cELISA-2 in [20]). The details of the method are provided in S1 Text.

### Animal ethics

The collection of rabbits from national parks was covered by the scientific licence #12226/2007 issued by the New South Wales department of Environment and Climate Change (for Cattai National Park), licenses Q25952-1, Q25952-2 and Q25952-3 issued by the South Australian Department for Environment, Water and Natural Resources (national parks in South Australia), or collected by/with permission of individual parks rangers as part of routine pest animal management. The majority of the sampling was carried out on private land with the landholder's permission, and the authors should be contacted for potential future sampling at these sites. Rabbits are a declared pest species in Australia, and no specific permission was required to collect rabbits with the exceptions listed above. The field studies did not involve any endangered or protected species. The specific locations of all sampling sites are listed in S1 Table.

Rabbits were either shot or trapped. Shooting was carried out by licensed shooters with a.22 calibre rifle according to Standard Operating Procedures for Ground Shooting of Rabbits. Trapping was carried out using cage paddle traps baited with carrots or oats. Traps were set at dusk and checked within two hours of sunrise, rabbits were removed from the traps and killed by cervical dislocation. All efforts were made to minimise suffering. The methods involving sample collection from rabbits were carried out according to the Australian code for the care and use of animals for scientific purposes and covered by the following AEC Permit numbers: 23/98, 10/99, 13/01, 09/03 and 11/09 issued by the Department of Primary Industries and Regions South Australia Animal Ethics Committee, permit number VPRU ORA 11-14-001 issued by the New South Wales Department of Primary Industries Orange Animal Ethics Committee, permit number 95/96-21, issued by the CSIRO Wildlife and Ecology Animal Experimentation Ethics Committee, permit numbers CSEAEC # 06–31, CESAEC # 09–14, CESAEC # 12–15 issued by the Commonwealth Scientific and Industrial Research Organisation (Sustainable Ecosystems and Ecosystem Sciences Division) Animal Ethics Committees, permit numbers Q25952-1 and Q25952-2 issued by the Department of Environment Water and Natural Resources Animal Ethics Committee, PAEC 060601, SAWEC 45/2007 and CA2013/01/662 issued by Queensland Government animal ethics committees.

### Statistical model

We used generalised linear mixed-effects models (GLMM; Binomial distribution) to identify climatic predictors (temperature and rainfall) of RCV-A1 prevalence (i.e., proportional occurrence of RCV-A1 positive sera at each site). Since mean annual rainfall can vary considerably between years, and wild rabbits rarely live beyond three years of age [21], we modelled climate predictors as 3-year rainfall and temperature averages of the sampling year and the proceeding 2 years. Climate variables were 3-year average minimum temperature (*Tmin*), 3-year average maximum temperature (*Tmax*) and 3-year average rainfall (*Rain*). Variable choice was guided by expert opinion on the most likely climatic drivers of RCV-A1 prevalence [7], [10]. All daily climate data were obtained from the Australian Government Bureau of Meteorology for the weather station closest to the coordinates of each sampling site.

The two-vector response variable comprised the number of screened animals that were positive for RCV-A1 antibody and the number of animals that were negative at each site in each year based on the serological assays. *Year* was treated as a random effect, not a fixed effect, because we were interested in the variation among years and not the specific effect of year [22]. Based on likelihood ratio tests [23], there was no support for treating *site* as an additional random effect. To avoid co-linearity among predictors, climatic variables with a Spearman's Rank correlation ≥0.7 were excluded from the analysis. *Tmin* and *Tmax* were strongly correlated so we removed *Tmax* but retained *Tmin* because it was the least correlated with *Rain*.

We explored the influence of climate on RCV-A1 prevalence using a candidate model set including *Tmin*, *Rain* and log transformed *Rain* (*logRain*); and some combinations of these (Table 1). We did not model quadratic or interaction terms because doing so resulted in models not converging or large standard errors for coefficient estimates for fixed effects. We compared and ranked models using Akaike's information criterion corrected for finite sample size (AIC_c_) and the dimension-consistent Bayesian information criterion (BIC; an approximation of the Bayes factor given no informative prior information on relative model support [24]). Specifically, we used BIC model weights (wBIC) to determine the contribution of the most important variables and AIC weights (wAIC_c_) to identify the most useful predictive models given the data [25]. We assessed each model's structural goodness-of-fit with per cent deviance explained (%DE). Observation-level random effects were fitted to the models to account for issues of overdispersion (σ = 1.8) [26]. Likelihood ratio tests were used to confirm support for modelling an observational level random effect. All residual distributions met linear model assumptions (assessed using normalised scores of standardised residual deviance [Q-Q plots]). All models were implemented using lme4 package in Program R [27].

**Table 1:** Correlates of RCV-A1 prevalence in wild rabbit populations in Australia.

Model	k	LL	dAIC_c_	wAIC_c_	dBIC	wBIC	% DEV
Tmin^+^logRain^+^(1|year)^+^(1|obs)	6	−117.04	0.00	1.00	0.00	1.00	25.79
logRain^+^(1|year)^+^(1|obs)	5	−130.84	25.31	0.00	23.06	0.00	17.04
Tmin^+^(1|year)^+^(1|obs)	5	−132.78	29.19	0.00	26.95	0.00	15.81
Rain^+^(1|year)^+^(1|obs)	5	−138.70	41.02	0.00	38.77	0.00	12.06
null	4	−157.72	76.84	0.00	72.29	0.00	0.00

This table showed number of parameters (k), Log-likelihood (LL), change in AICc compared with the best-ranked model (dAICc), model AICc weights (wAICc), change in BIC compared with the best-ranked model (dBIC), model BIC weight (wBIC) and per cent deviance explained (% DEV). Climate variables are 3-year averages: annual min temperature (*Tmin*), annual rainfall (*Rain*) and log annual rainfall (*logRain*). We modelled year as random effect. An observation-level random effect (obs) was used to account for issues of overdispersion. The null model has only random effects. See Methods for further details.

Spatial correlograms were generated using Moran's I to assess autocorrelation in RCV-A1 prevalence (raw data and GLMM residuals) as a function of the distance between sites [28]. Evidence for spatial autocorrelation was assessed after a Bonferroni correction [29]. The analysis was implemented using the *spdep* package in Program R.

We assessed the predictive capacity of the most parsimonious model (i.e., with greatest AICc support) using standard 10-fold cross-validation, reporting mean absolute prediction error for the response variable [30]. This model was used to make spatial predictions (at a grid cell resolution of ∼5 km) of the probability of RCV-A1 occurrence for the present period of high rainfall (3-year average, focused on 2011) and a historic period of drought conditions (3-year average, focused on 2003). The Southern Oscillation Index (SOI) which is a measure of fluctuations in the air pressure difference between Tahiti and Darwin, Australia was used for selection of wet and dry periods [31]. Sustained negative values of the SOI below −8 (El Niño events, as was observed between 2002 and 2004) are often associated with a reduction in rainfall over much of eastern and northern Australia. Conversely, positive values of the SOI above +8 (La Niña events, as was observed between 2010 and 2012) increase the probability that eastern and northern Australia will receive above-average rainfall.

Annual climate prediction surfaces for Australia were accessed from the Australian Government Bureau of Meteorology: average annual minimum temperature and rainfall (0.05×0.05° grid cell resolution) for the years 2002–2004 and 2010–2012. Interpolated data were generated using an optimised Barnes successive correction technique that applies a weighted averaging process to the weather station data from across Australia [32]. We constrained our model predictions to climate conditions used to build the model. By doing this, we avoided using the model to extrapolate to novel climatic conditions, because this can result in increased levels of uncertainty in model predictions [33].

## Results

### Distribution of RCV-A1 in Australia

**Figure 1:**
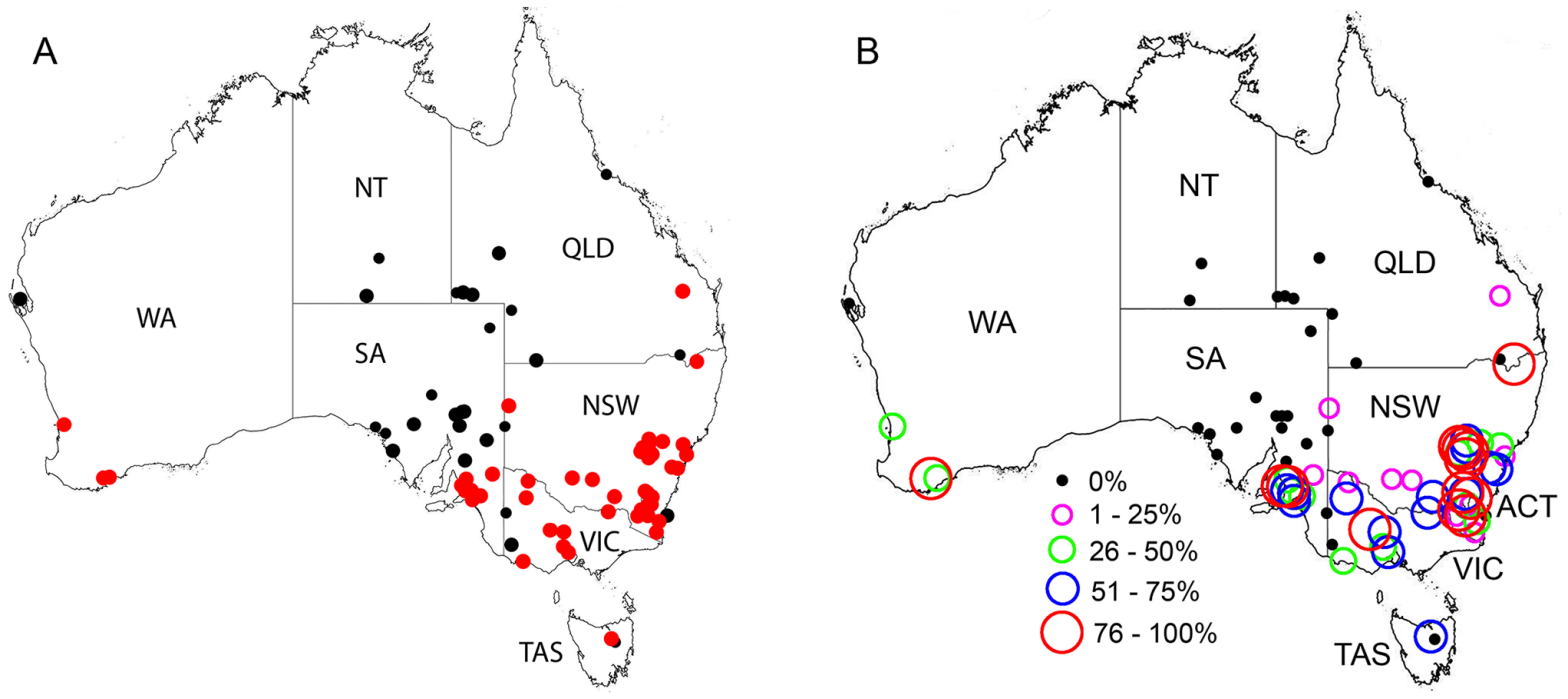
The distribution and prevalence of RCV-A1 in wild rabbit populations in Australia. (A) The presence (red dot) and absence (black dot) of RCV-A1 at different sites. Black dots at smaller size indicate sites with sample numbers less than 20. (B) Prevalence of RCV-A1 at the 78 sites tested. Black dot: prevalence of RCV-A1 is 0% in the tested samples. Circles with varied size and colour indicate the different prevalence of RCV-A1 at each site. WA: Western Australia, NT: Northern Territory, SA: South Australia, QLD: Queensland, NSW: New South Wales, ACT: Australian Capital Territory, VIC: Victoria, TAS: Tasmania. The maps were generated using the “Mapping and analysis” tool from the ‘Atlas of Living Australia’ (http://www.ala.org.au/), licensed under a Creative Commons Attribution 3.0 Australia License.

Antibodies to RCV-A1 were confirmed in rabbit populations from 53 of the 78 sites tested. Where present, the sero-prevalence of RCV-A1 in different rabbit populations varied from 13% to 100% (S1 Table). RCV-A1 was found on mainland Australia in relatively narrow strips along the south-eastern and south-western coastlines and in Tasmania (TAS) (Fig. 1A). When the prevalence of RCV-A1 was grouped into five categories from low to high, the sites with higher prevalence were largely clustered in the south-east of New South Wales (NSW) and Victoria (VIC), and the Mount Lofty Ranges near Adelaide in South Australia (SA) (Fig. 1B).

No evidence of RCV-A1 antibodies was found at sites located in arid parts of the Northern Territory (NT), Western Australia (WA), Queensland (QLD) and the north of SA. The only site in NSW where rabbits tested negative to RCV-A1 was Montague Island which is isolated from the mainland. The two negative sites in VIC were interspersed with positive sites, and the sample from one of the sites was small (*n* = 6), and therefore overall confidence in the negative observation in VIC is reduced.

The 3-year average annual rainfall at sites where RCV-A1 antibodies were detected was 573±217 mm (mean ±SD), compared to 308±200 mm (mean ±SD) at sites where RCV-A1 antibodies were not detected. Sites with high prevalence of RCV-A1 (>50%) had a mean annual rainfall of 608±179 mm (mean ±SD).

### Correlates of RCV-A1 prevalence

There was strong statistical support for RCV-A1 prevalence as a function of temperature and rainfall, which was best explained using a multi-term model with *Tmin* and *logRain* (Table 1). This model explained 26% of model structural deviance. There was no support for the next ranked model, which treated the proportional occurrence of RCV-A1 as a function of *logRain* (dAICc = 25.31, dBIC = 23.06, Dev = 17%). The model averaged coefficients (fixed effects) for the most parsimonious model were: *Tmin*  = −0.298 (SE = 0.032); *logRain*  = 2.21 (SE = 0.205).

Cross-validated mean prediction error according to the most parsimonious model (i.e., ∼ *Tmin* + *logRain* + [1|year] + [1|obs])) for RCV-A1 prevalence was ±0.199. There was no evidence of spatial autocorrelation in GLMM residuals (i.e. *P*>0.05 at all distance classes).

#### Projection of RCV-A1 prevalence under different climate scenarios

**Figure 2:**
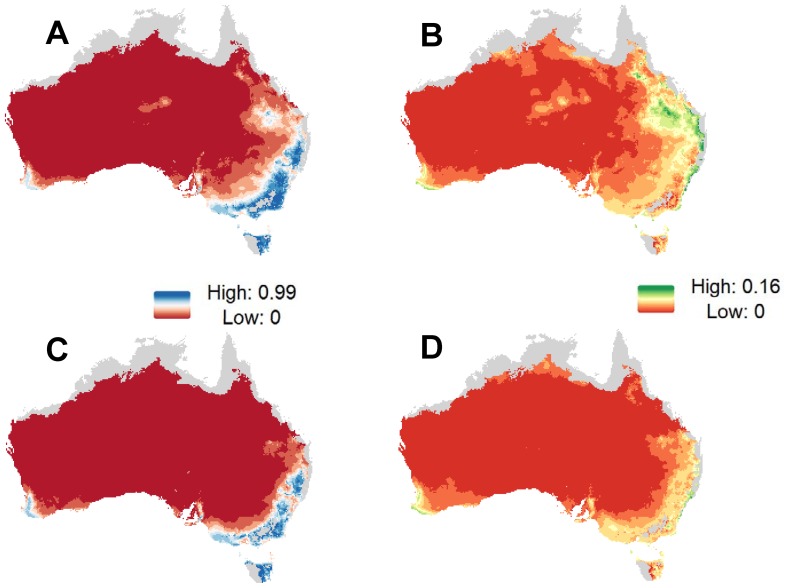
Predicted RCV-A1 prevalence in Australia and associated standard error for wet and dry periods. (A) and (B) RCV-A1 prevalence and standard error for an episode of above average rainfall from 2010 to 2012 (Wet Period). (C) and (D) RCV-A1 prevalence and standard error for an episode of below average rainfall from 2002 to 2004 (Dry Period). Areas in grey represent novel climate conditions, where predictions could not be made.

**Figure 3:**
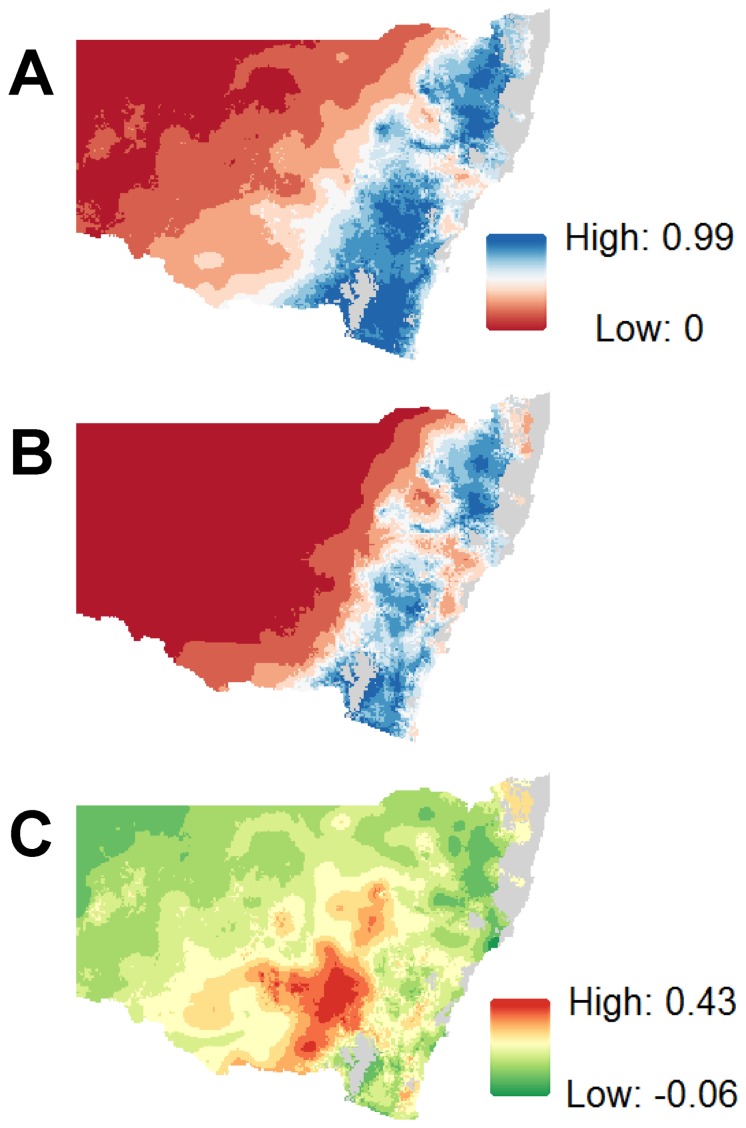
Predicted RCV-A1 prevalence in New South Wales, Australia. (A) RCV-A1 prevalence for a period (2010–2012) of above average rainfall. (B) RCV-A1 prevalence for a period (2002–2004) of below average rainfall. (C) The grid-cell difference between predicted prevalence in a wet year and a dry year.

This model was used to predict RCV-A1 prevalence for observed periods of wet and drought conditions based on rainfall and minimum temperature data, i.e. 3-year average focused on 2011 and 2003, respectively. It revealed that: (i) RCV-A1 prevalence was highest along the east coast of Australia, Tasmania, the Fleurieu Peninsula and Mt Lofty regions of South Australia, and the southern corner of Western Australia; and (ii) drought conditions negatively impacted the distribution of RCV-A1 (Fig. 2). During above average wet years in NSW (a region of eastern Australia) RCV-A1 prevalence increased in a westerly direction i.e., encroaching into typically more arid regions of Australia where it was not found during periods of drought (Fig. 3A and B). RCV-A1 prevalence increased by up to 0.43 in some areas of NSW in response to higher-than-average rainfall (Fig. 3C).

## Discussion

We describe here for the first time a comprehensive map of the distribution and prevalence of the benign calicivirus RCV-A1 across the Australian continent. We show evidence of a strong correlation between RCV-A1 prevalence and temperature and rainfall, and propose that the prevalence and distribution of RCV-A1 may vary in response to short-term changes in climatic conditions, thereby providing a valuable new tool for predicting the dynamics of RCV-A1 at continental and regional scales in the context of climate change. By improving understanding of the potential causes of decreased effectiveness of RHDV in cooler wetter areas in Australia, our results can be used when planning the future release of new pathogenic caliciviruses and integrating non-biological methods in these areas for rabbit control.

### Correlation between RCV-A1 prevalence and climate factors

RCV-A1 has likely existed in Australia for 150 years [17]. Thus, the distribution of this established virus should have reached a dynamic equilibrium in rabbit populations. It was therefore appropriate to use a modelling approach to predict RCV-A1 distribution at a continental scale. We showed that a model with temperature and rainfall (i.e. *Tmin* and and *logRain*) can explain up to 26% variance in RCV-A1 prevalence; and that the distribution of RCV-A1 is likely to be restricted to areas with cooler and more humid climates, including the cooler part of sub-tropical Queensland. In doing so we provided important evidence to support earlier hypotheses that RCV-A1 is likely to be well established in wild rabbit populations in cool, wet areas of Australia [7], [18]. We also propose that, according to this model, the prevalence of RCV-A1 is likely to vary spatio-temporally in response to short to medium-term variation in rainfall.

The strong association between the occurrence of RCV-A1 and climate factors is at odds with the distribution of the pathogenic RHDV, which has been able to spread naturally into all rabbit populations including those in the hot arid and semi-arid inland [4], [5]. Closer examination of epidemiological factors such as the relative titres of virus produced and mode of virus transmission may provide the most likely explanation for the mechanisms by which climate could affect virus spread. RHDV grows to very high titres [15], [34] and while it can spread via the direct faecal-oral route [35], the high virus load in the carcasses also enables mechanical transmission through flies over long distances [36]–[39]. In contrast, the low virus titre in tissues and excretions of RCV-A1 infected rabbits [14] makes the transmission of RCV-A1 by insects unlikely. The localized geographic distribution of RCV-A1 in wild rabbit populations [17] and the spread of RCV-A1 in a rabbit colony [16] support a direct contact or close proximity transmission model for RCV-A1. While RCV-A1 can be detected in the bile months after infection [15], true persistent infections and reactivation of virus shedding have not been confirmed. However, it is feasible that a constant supply of susceptible rabbits is sufficient to allow RCV-A1 to persist by continual transmission, for example, in environments that favour prolonged breeding of rabbits throughout the year and support high rabbit densities. Long breeding seasons supporting the persistence of viruses in rabbits has also been demonstrated for myxoma virus [40].

In this respect, it is important to note that duration and frequency of rabbit breeding are strongly influenced by available soil-moisture, itself a product of rainfall and evapo-transpiration [41]. Meanwhile, evapo-transpiration is largely driven by temperature. The sites with higher prevalence of RCV-A1 are concordant with areas where rainfall is consistently high throughout the year and favours a long breeding season of rabbits. Therefore, it is highly likely that the strong correlation between RCV-A1 and climate is unlikely to be a direct response to climate *per se*. Rather; the climatic variables identified to correlate with high prevalence of RCV-A1 are likely to influence breeding patterns and rabbit densities, which in turn may support efficient spread of RCV-A1. However, even within areas with generally high prevalence of RCV-A1, the distribution of RCV-A1 may be patchy, possibly due to different fine-scale climate conditions, and other factors such as rabbit control programs using poisons may reduce rabbit densities locally, irrespective of climatic factors.

### RCV-A1 impacts the effectiveness of RHDV

RCV-A1 can decrease the mortality of rabbits following RHDV infections [7], [9], [12], [14], [16], [42]. For example, sera sampled before the first RHDV release in 1996 in NSW (see Cattai National Park, S1 Table) showed high RCV-A1 prevalence. Indeed, there was no evidence of RHDV spread or reduction in rabbit numbers after two deliberate RHDV releases [43]. Furthermore, rabbits as young as three weeks have been found infected with RCV-A1 (T. Strive, unpublished data), so it is possible that most rabbits are infected with RCV-A1 before they are exposed to RHDV in areas with high prevalence of RCV-A1.

In areas with low prevalence of RCV-A1, the impact of RCV-A1 on RHDV effectiveness may not be obvious after the initial spread of RHDV, but with even a small proportion of rabbits surviving the RHDV challenge due to pre-infection with RCV-A1 each year, the high reproduction rate of these life-long immune rabbits may contribute significantly to the long-term recovery of the population.

It should be noted that the climatic conditions associated with high RCV-A1 prevalence can also favour year round breeding of rabbit populations. For reasons that are not completely understood, RHDV case fatality is greatly reduced in very young rabbits [44]. The observed reduction of RHDV effectiveness in these areas may therefore a result of the combined effects of high RCV-A1 prevalence as well as the age structure of the population.

### Impacts of RHDV on RCV-A1 prevalence

While RCV-A1 can directly reduce the impact of RHDV epidemic, it is also possible that RHDV can indirectly reduce the prevalence of RCV-A1. In the case of RCV-A1 which is transmitted mainly by the fecal-oral route, it is likely that the fragmentation of rabbit populations is a major handicap to the re-colonization of the virus in patchy rabbit populations. As a case in point, after the initial RHDV release in 1996, rabbit population size declined drastically in south-western Australia [45], meanwhile RCV-A1 prevalence changed dramatically from 87% to 0% in this area (Stirling Ranges) between 1993 and 2012 (S1 Table; T. Cox, unpublished data). Furthermore, in semi-arid areas of western NSW (Broken Hill) and SA (Manunda), RCV-A1 prevalence reduced from 60% to 0% between 1995 (before RHD outbreaks) and 2012 (S1 Table). Both the introduction of RHDV and degeneration of vegetation across the area due to a decrease in late autumn and winter rainfall across southern Australia since 1974 [46] have likely contributed to the reduction in rabbit density, which in turn may have led to regional extinction of RCV-A1. However, such a reduction in population sizes followed by an apparent drop in RCV-prevalence was only observed at semi-arid sites located at the edges of RCV-A1 distribution. In coastal areas where climate permits year round breeding of rabbits, RHDV had very little impact on rabbit densities, and RCV-A1 prevalence has remained high [43]. More research is needed to test whether RHDV or the change in climate or both were the main driver for this drastic change of RCV-A1 prevalence. However, if rabbit density is the limiting factor of RCV-A1 persistence in these areas, it may be equally feasible that RCV-A1 could spread back into these semi-arid areas if rabbit numbers were allowed to build up again.

### Implications for rabbit management in Australia

In order to maximize the benefits of RHDV as a biological control agent and justify the cost of any additional control methods, integrated management plans with long-term economic and environmental strategies are needed. The presence of RCV-A1 in wild rabbit populations in Australia complicates rabbit management and considerations of how to best use RHDV as a cost-effective bio-control agent. Although the protection against lethal RHDV infection by pre-infection of RCV-A1 is transient [16] and possible seasonal patterns of RCV-A1 activity are currently unknown, efforts to initiate fresh RHDV outbreaks may have little impact in areas of high RCV-A1 prevalence, and alternative conventional control methods such as warren ripping [47] should be considered. In contrast, in areas with zero or low RCV-A1 prevalence, additional RHDV releases may prove a cost effective tool for rabbit management, provided they are well timed and applied when few very young rabbit kittens are present in the population, and the proportion of adult rabbits with RHDV antibodies is low.

Ideally, the site-specific presence and prevalence of RCV-A1 should be known prior to the release of RHDV, but since this is not likely to be practicable in most circumstances, the predicted RCV-A1 prevalence map can be a powerful tool to aid decision making by pest managers once its accuracy is demonstrated by sampling and testing for RCV-A1 seroprevalence at representative sites. Furthermore, the predictive model can be progressively updated with new climate data from each year, to predict if and how RCV-A1 distribution and prevalence are likely to change, and rabbit management strategies that rely on RHDV can be adjusted accordingly. For example, if RCV-A1 moves to the west in wet years, the choice for rabbit control in these areas may be changed in a timely fashion from release of RHDV to other control methods. Or if, under climate change, existing wet areas become appreciably drier, as they already have in south-west WA, the cost for rabbit control may be reduced by targeted RHDV releases.

In contrast, the wild rabbit is a key-stone species of some ecosystems in Europe and rabbit conservation is needed due to the challenge imposed on rabbits by RHDV and predators [48], [49]. A number of benign rabbit caliciviruses affording different levels of cross protection against RHDV have been recorded in Europe in areas where RHDV is endemic [50]–[52], however the detailed epidemiological patterns of these viruses in wild populations are currently not well understood. The approach presented in this study may prove useful to gain better understanding of the distribution and prevalence of other non-pathogenic enteric caliciviruses in Europe, and in New Zealand where the presence of benign caliciviruses have yet to be confirmed.

## Supporting Information

S1 Table **The prevalence of RCV-A1 antibodies at different sites.**
(DOCX)

S1 Text **Details of the serological assay used in this study.**
(DOCX)
