# Supplementary material for: Distribution and Prevalence of the Australian Non-Pathogenic Rabbit Calicivirus Is Correlated with Rainfall and Temperature
Source: PLoS One. 2014 Dec 8;9(12):e113976. doi: 10.1371/journal.pone.0113976 (PMC4259302; doi:10.1371/journal.pone.0113976)
Supplement: S1 Table — The prevalence of RCV-A1 antibodies at different sites. (DOCX) [file pone.0113976.s001.docx]

**Supporting information PONE Liu et al., 2014**

**Table S1.** The prevalence of RCV-A1 antibodies at different sites. The data is organized according to the prevalence of RCV-A1 from low to high.

| **Site ID** | **Site name** | **State** | **Longitude** | **Latitude** | **Year of sampling** | **No. of sera** | **Prevalence of RCV-A1 (%)** | **Annual mean rainfall* (mm)** | **Mean Tmin* (degree)** |
| --- | --- | --- | --- | --- | --- | --- | --- | --- | --- |
| **1** | Montague Island | NSW | 150.22704 | -36.253017 | 1995 | 20 | 0 | 885 | 13.7 |
| **2** | Alice Springs | NT | 133.88124 | -23.686935 | 2007 | 16 | 0 | 205 | 14 |
| **3** | Erldunda Station | NT | 133.17772 | -25.611715 | 1994 | 21 | 0 | 195 | 14.1 |
|  | Erldunda Station | NT | 133.17772 | -25.611715 | 2011 | 29 | 0 | 364 | 13.7 |
| **4** | Barcoo Shire | QLD | 141.41667 | -26.333332 | 1995 | 17 | 0 | 178 | 16 |
| **5** | Diamantina Shire | QLD | 139.1905 | -25.544899 | 1995 | 20 | 0 | 152 | 15.8 |
| **6** | East Simpson Desert | QLD | 138.27391 | -25.449528 | 1995 | 17 | 0 | 152 | 15.8 |
| **7** | Bulloo Downs | QLD | 142.80942 | -28.851849 | 2008 | 30 | 0 | 180 | 15 |
| **8** | Diamantina Lakes | QLD | 140.68636 | -23.420715 | 2007 | 25 | 0 | 174 | 18.7 |
| **9** | Townsville | QLD | 146.80109 | -19.259003 | 2007 | 10 | 0 | 934 | 20.2 |
| **10** | Muncoonie Lakes | QLD | 138.65411 | -25.43616 | 1995 | 20 | 0 | 152 | 15.8 |
|  | Muncoonie Lakes | QLD | 138.65411 | -25.43616 | 2011 | 15 | 0 | 365 | 16.5 |
| **11** | Flinders Ranges | SA | 138.23721 | -31.509277 | 1994 | 20 | 0 | 204 | 12.3 |
| **12** | Ceduna | SA | 133.68971 | -32.107006 | 1996 | 16 | 0 | 240 | 10.3 |
| **13** | Kooma | SA | 134.2579 | -32.42377 | 1996 | 17 | 0 | 273 | 12.3 |
| **14** | Gawler Ranges | SA | 135.867 | -31.97177 | 1996 | 29 | 0 | 163 | 10 |
| **15** | Roxby Downs | SA | 136.88503 | -30.54763 | 1996 | 8 | 0 | 156 | 13.1 |
| **16** | Gum Creek | SA | 138.7561 | -33.705082 | 1999 | 20 | 0 | 447 | 8.8 |
| **17** | Yednalue | SA | 138.4588 | -32.045128 | 2001 | 20 | 0 | 361 | 11.4 |
| **18** | Yanyanna Track | SA | 138.6174 | -31.368162 | 2003 | 20 | 0 | 198 | 10.9 |
| **19** | Coongie lakes | SA | 140.17152 | -27.232292 | 2007 | 19 | 0 | 171 | 15.9 |
| **20** | Venus Bay | SA | 134.68079 | -33.234535 | 2001 | 20 | 0 | 409 | 12.1 |
|  | Venus Bay | SA | 134.68079 | -33.234535 | 2003 | 20 | 0 | 407 | 11.7 |
| **21** | Oraparinna | SA | 138.73055 | -31.349821 | 2003 | 10 | 0 | 198 | 10.9 |
|  | Oraparinna | SA | 138.73055 | -31.349821 | 2005 | 10 | 0 | 236 | 11.1 |
|  | Oraparinna | SA | 138.73055 | -31.349821 | 2008 | 10 | 0 | 180 | 10.9 |
|  | Oraparinna | SA | 138.73055 | -31.349821 | 2011 | 10 | 0 | 286 | 11.2 |
| **22** | Epping Forest | TAS | 147.3151 | -41.800186 | 2012 | 9 | 0 | 616 | 6 |
| **23** | Casterton | VIC | 141.40973 | -37.58122 | 1972 | 20 | 0 | 496 | 8.4 |
| **24** | Telopea Downs hall | VIC | 141.10846 | -36.126156 | 1996 | 6 | 0 | 399 | 7.2 |
| **25** | Shark Bay | WA | 113.54672 | -25.763716 | 2008 | 22 | 0 | 190 | 16.2 |
| **26** | Whetstone | QLD | 150.95023 | -28.584194 | 1995 | 20 | 20 | 568 | 11 |
|  | Whetstone | QLD | 150.95023 | -28.584194 | 2007 | 16 | 0 | 483 | 12.4 |
| **27** | Hattah-Kulkyne National Park | VIC | 142.35571 | -34.662327 | 2011 | 15 | 13 | 417 | 10.5 |
| **28** | Fowler's gap | NSW | 141.25761 | -31.081242 | 1996 | 14 | 14 | 171 | 12 |
| **29** | Tinderry Ranges | NSW | 149.33499 | -35.740677 | 2007 | 7 | 14 | 504 | 7.2 |
| **30** | Eidsvold and Munduberra | QLD | 151.11751 | -25.375381 | 1995 | 22 | 14 | 558 | 14.1 |
| **31** | Burragate | NSW | 149.62083 | -37.019024 | 2007 | 6 | 17 | 730 | 9.8 |
| **32** | Darlington Point | NSW | 145.98595 | -34.593933 | 2002 | 20 | 20 | 364 | 10 |
| **33** | Kosciiusko | NSW | 148.56535 | -36.260708 | 2003 | 15 | 20 | 522 | 10.2 |
| **34** | Mallee | VIC | 140.31381 | -34.334446 | 1996 | 20 | 20 | 247 | 8.1 |
| **35** | Hay | NSW | 144.84445 | -34.508965 | 2002 | 20 | 25 | 289 | 10.6 |
| **36** | Gosford | NSW | 151.333 | -33.417 | 2003 | 8 | 25 | 1275 | 11.2 |
| **37** | Monarto Zoo | SA | 139.11496 | -35.081974 | 2004 | 15 | 27 | 336 | 9.6 |
| **38** | Rylstone | NSW | 149.967 | -32.8 | 2002 | 20 | 30 | 669 | 8.2 |
| **39** | Yambuk | VIC | 142.06453 | -38.315655 | 2007 | 19 | 32 | 694 | 10.4 |
| **40** | Cudal | NSW | 148.73584 | -33.2868 | 2000 | 3 | 33 | 800 | 7.5 |
| **41** | Michelago | NSW | 149.15501 | -35.74184 | 2007 | 66 | 33 | 504 | 7.2 |
| **42** | Perth | WA | 116.030945 | -31.98697 | 1993 | 6 | 33 | 1117 | 12.8 |
| **43** | Bacchus Marsh | VIC | 144.33658 | -37.65374 | 2007 | 61 | 37 | 550 | 8.5 |
| **44** | Wollombi | NSW | 151.13893 | -32.940777 | 2002 | 18 | 39 | 714 | 11.3 |
| **45** | Turretfield | SA | 138.82297 | -34.56174 | 2010 | 13 | 31 | 510 | 10 |
|  | Turretfield | SA | 138.82297 | -34.56174 | 2011 | 14 | 64 | 539 | 10.1 |
| **46** | Manunda in Flinders District | SA | 139.7217 | -32.768772 | 1995 | 20 | 65 | 258 | 6.7 |
|  | Manunda in Flinders District | SA | 139.7217 | -32.768772 | 1997 | 20 | 20 | 248 | 7.3 |
|  | Manunda near Yunta | SA | 139.7217 | -32.768772 | 2012 | 2 | 0 | 201 | 9.8 |
|  | Melton's Station near Manunda | SA | 139.28152 | -32.24461 | 2012 | 24 | 0 | 388 | 9.8 |
| **47** | Coorong | SA | 139.64928 | -35.337177 | 2011 | 20 | 45 | 431 | 9.4 |
| **48** | Wellstead, Green Range | WA | 118.60426 | -34.477135 | 1995 | 20 | 45 | 470 | 9.6 |
| **49** | Brogo | NSW | 149.78395 | -36.518673 | 2007 | 30 | 47 | 886 | 8.7 |
| **50** | Nurrung Peninsula | SA | 139.182 | -35.513 | 2001 | 12 | 33 | 451 | 10.7 |
|  | Nurrung Peninsula | SA | 139.182 | -35.513 | 2002 | 7 | 86 | 452 | 10.5 |
|  | Nurrung Peninsula | SA | 139.182 | -35.513 | 2003 | 1 | 100 | 450 | 10.1 |
| **51** | Werribee | VIC | 144.6398 | -37.92174 | 1972 | 20 | 55 | 542 | 9.1 |
| **52** | Echunga | SA | 138.79633 | -35.103592 | 2004 | 16 | 56 | 746 | 8.3 |
| **53** | Albury | NSW | 146.90222 | -36.06393 | 2000 | 17 | 59 | 788 | 9.3 |
| **54** | Broken Hill | NSW | 141.04353 | -32.085262 | 1996 | 20 | 60 | 240 | 10.6 |
|  | Broken Hill (Thackaringa) | NSW | 141.04353 | -32.085262 | 2012 | 19 | 0 | 315 | 11.5 |
| **55** | Cattai | NSW | 150.48967 | -34.002167 | 2007 | 26 | 62 | 846 | 10.4 |
| **56** | Gungahlin | ACT | 149.12396 | -35.22069 | 2007 | 8 | 88 | 566 | 7.5 |
|  | Gungahlin | ACT | 149.12396 | -35.22069 | 2008 | 17 | 65 | 510 | 7.5 |
|  | Gungahlin | ACT | 149.12396 | -35.22069 | 2010 | 34 | 59 | 749 | 7.7 |
| **57** | Campbelltown | NSW | 150.817 | -34.067 | 2002 | 36 | 64 | 540 | 10.1 |
| **58** | Longford | TAS | 147.11002 | -41.648193 | 2012 | 14 | 64 | 727 | 6 |
| **59** | Wagga, Mangoplah | NSW | 147.2663 | -35.371487 | 2002 | 20 | 65 | 514 | 9.5 |
| **60** | Euchareena | NSW | 149.19023 | -32.97956 | 2011 | 29 | 69 | 953 | 6.6 |
| **61** | Pine Plains | VIC | 142.23553 | -35.437664 | 1976 | 22 | 73 | 378 | 9.8 |
| **62** | Burrendong | NSW | 149.19954 | -32.70064 | 1997 | 20 | 75 | 609 | 9.5 |
| **63** | Murray Bridge | SA | 139.22122 | -35.23082 | 1996 | 20 | 75 | 299 | 9.5 |
| **64** | Bendigo | VIC | 144.36815 | -37.00406 | 2009 | 48 | 75 | 480 | 7.8 |
| **65** | Hall | NSW | 148.97882 | -35.12224 | 2007 | 15 | 80 | 586 | 7.5 |
| **66** | Stirling Ranges | WA | 118.251274 | -34.5215 | 1996 | 1 | 0 | 528 | 10.2 |
|  | Stirling Ranges | WA | 118.251274 | -34.5215 | 1998 | 15 | 87 | 653 | 10.4 |
|  | Stirling Ranges | WA | 118.251274 | -34.5215 | 1999 | 4 | 75 | 680 | 10.5 |
| **67** | Oaky Creek | NSW | 149.21 | -33.24 | 2007 | 21 | 71 | 685 | 7.5 |
| **68** | Dunluce | VIC | 143.59265 | -36.908848 | 1999 | 15 | 87 | 447 | 7.9 |
|  | Dunluce | VIC | 143.59265 | -36.908848 | 2000 | 6 | 83 | 497 | 8.1 |
| **69** | Carwoola | NSW | 149.37975 | -35.39349 | 2010 | 17 | 88 | 664 | 7.3 |
| **70** | Adaminaby | NSW | 148.76793 | -35.98316 | 2002 | 19 | 89 | 586 | 4.2 |
| **71** | Lenswood | SA | 138.82893 | -34.919853 | 2003 | 18 | 89 | 865 | 8.2 |
| **72** | Molong | NSW | 148.85 | -33.1 | 2003 | 10 | 90 | 592 | 7.4 |
| **73** | Stanthorpe | QLD | 151.92099 | -28.921787 | 2006 | 52 | 92 | 801 | 7.2 |
| **74** | Cooma | NSW | 149.13954 | -36.238033 | 1994 | 20 | 95 | 541 | 3.6 |
| **75** | Valpine | NSW | 149.372 | -33.403164 | 2007 | 26 | 88 | 622 | 6.5 |
| **76** | Carcoar | NSW | 149.19315 | -33.573784 | 2000 | 20 | 95 | 984 | 6.2 |
| **77** | Mullion Creek | NSW | 149.117 | -33.133 | 2002 | 20 | 100 | 801 | 7.4 |
| **78** | Belair National Park | SA | 138.66 | -35 | 2003 | 20 | 100 | 684 | 8.2 |

* Average rainfall or temperature in the proceeding 2 years and the year of sampling.
