# Supplementary material for: Distribution and Prevalence of the Australian Non-Pathogenic Rabbit Calicivirus Is Correlated with Rainfall and Temperature
Source: PLoS One. 2014 Dec 8;9(12):e113976. doi: 10.1371/journal.pone.0113976 (PMC4259302; doi:10.1371/journal.pone.0113976)
Supplement: S1 Text — Details of the serological assay used in this study. (DOCX) [file pone.0113976.s002.docx]

**Supporting information**

**Text S1: Details of the serological assay used in this study.**

After coating ELISA plates with chicken anti-RCV-A1 polyclonal antibodies, antigen RCV-A1-VLP (virus-like-particle) was added and incubated. After washing, rabbit serum was added at dilutions of 1:10, 1:40 and 1:160 in duplicate, followed immediately by the addition of anti-RCV-A1 specific mouse monoclonal antibody. Goat anti-mouse IgG-horseradish peroxidase (Abcam, Cambridge) was used for detection. The optical density at 492 nm (OD492) of negative serum at each dilution was defined as 100%, and rabbit serum with more than 25% reduction in OD492 was scored as positive. The percentage of sero-positive samples in each population was used to estimate the prevalence of RCV-A1 antibodies for the respective site. A detailed description of the method can be found in [20].
